# Synthesis of novel multifunctional carbazole-based molecules and their thermal, electrochemical and optical properties

**DOI:** 10.3762/bjoc.16.93

**Published:** 2020-05-19

**Authors:** Nuray Altinolcek, Ahmet Battal, Mustafa Tavasli, William J Peveler, Holly A Yu, Peter J Skabara

**Affiliations:** 1 Department of Chemistry, Faculty of Science-Art, Uludag University, 16059 Nilufer, Bursa, Turkey; 2 Department of Elementary School Education, Faculty of Education, Mus Alparslan University, 49100, Mus, Turkey; 3 WestCHEM, School of Chemistry, University of Glasgow, Joseph Black Building, G128QQ Glasgow, UK

**Keywords:** carbazole, electrochemistry, fluorescence, formyl group, solvatochromism

## Abstract

Two novel carbazole-based compounds **7a** and **7b** were synthesised as potential candidates for application in organic electronics. The materials were fully characterised by NMR spectroscopy, mass spectrometry, FTIR, thermogravimetric analysis, differential scanning calorimetry, cyclic voltammetry, and absorption and emission spectroscopy. Compounds **7a** and **7b**, both of which were amorphous solids, were stable up to 291 °C and 307 °C, respectively. Compounds **7a** and **7b** show three distinctive absorption bands: high and mid energy bands due to locally excited (LE) transitions and low energy bands due to intramolecular charge transfer (ICT) transitions. In dichloromethane solutions compounds **7a** and **7b** gave emission maxima at 561 nm and 482 nm with quantum efficiencies of 5.4% and 97.4% ± 10%, respectively. At positive potential, compounds **7a** and **7b** gave two different oxidation peaks, respectively: quasi-reversible at 0.55 V and 0.71 V, and reversible at 0.84 V and 0.99 V. At negative potentials, compounds **7a** and **7b** only exhibited an irreversible reduction peak at −1.86 V and −1.93 V, respectively.

## Introduction

Carbazole derivatives have found many different applications in a variety of technologically important areas, such as organic light emitting diodes (OLEDs), organic photovoltaics (OPVs), dye synthesised solar cells (DSSCs) and sensors [1–2]. In OLEDs, carbazole derivatives are frequently used as host materials [3–5]. In this respect the most frequently used host materials are 1,3-bis(*N*-carbazolyl)benzene (mCP) [6] and polyvinylcarbazole (PVK) [7]. Carbazole derivatives, either just by themselves or in combination with iridium, are also used as emissive materials in OLEDs [8]. In this respect a molecule bearing a dithienylbenzothiadiazole unit and four alkyl-linked peripheral carbazole groups (named as TCTzC) is used in the construction of saturated red emissive OLEDs [9]. Carbazole-based homoleptic or heteroleptic iridium(III) complexes were also reported in the construction of different OLEDs [10–13]. In OPVs, carbazole derivatives are frequently used as small molecule p-type (electron-donating) materials or electron-accepting (n-type) materials with a variety of donor–acceptor combinations [14–15]. In sensor studies, carbazole derivatives are used as fluorophores. In this regard many different carbazole-based fluorophores are reported in the literature [16–19]. Some of the carbazole derivatives were used as colourimetric anion sensors [20], and others as biothiol sensors [21–23]. Research continues on carbazole derivatives to find new materials with novel properties. It is therefore essential that one should design a molecule that has multifunctional usage in many different areas of technology [24]. Since carbazole is a relatively inexpensive material with unique properties such as high hole-transporting mobility [12,25–27], pronounced thermal stability [2] and high fluorescent quantum yields [28–29] our attention was focused on carbazole derivatives. In addition to that, carbazole is a rigid aromatic molecule [30] with many different modification sites for multifunctionalisation [31].

In this work, we designed two novel 2-(*N*-hexylcarbazol-3’-yl)-4/5-formylpyridine compounds (**7a** and **7b**), where 4/5-pyridinecarboxyaldehyde was attached to the 3-position of carbazole via the 2-position of the pyridine ring. These two compounds (**7a** and **7b**) can be used in OLEDs, solar cells and sensor studies either directly or with small modifications. Here we report the synthesis, full characterisation and properties of these two novel compounds (**7a** and **7b**).

## Results and Discussion

### Synthesis

**Scheme 1:**
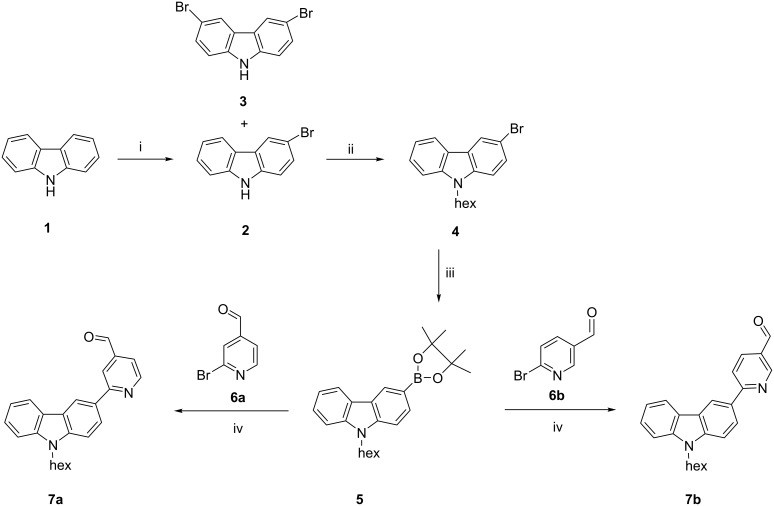
Synthesis of compounds **7a** and **7b** from carbazole **1**. i) NBS, DMF, 0 °C to rt, 24 h. ii) *n*-hexyl bromide, TBAI, NaOH (50%), 77 °C, 8 h. iii) B_2_(pin)_2_, KOAc, PdCI_2_(PPh_3_)_2_, 1,4-dioxane, 90 °C, 24 h. iv) **6a**/**6b**, K_2_CO_3_, PdCI_2_(PPh_3_)_2_, 90 °C, 6 h.

Compounds **7a** and **7b** were synthesised in four steps from carbazole (**1**) as depicted in Scheme 1. Carbazole (**1**) was first brominated with *N*-bromosuccinimide in dimethylformamide [32–33]. This gave a mixture of mono- and dibromo products **2** and **3**. Upon crystallisation 3-bromocarbazole (**2**) was obtained as white crystals in good yield. 3-Bromocarbazole (**2**) was then alkylated with 1-bromohexane in 50% aqueous NaOH in the presence of tetrabutylammonium iodide (TBAI) [33–35]. Upon chromatography 3-bromo-9-hexylcarbazole (**4**) was obtained as a liquid in good yield. 3-Bromo-9-hexylcarbazole (**4**) was then converted into the pinacol boronic ester by treating with bis(pinacolato)diboron in the presence of potassium acetate (KOAc) and dichlorobis(triphenylphosphine)palladium(II) in 1,4-dioxane [36–37]. Upon chromatography (9-hexylcarbazole-3-yl)boronic acid pinacol ester (**5**) was obtained as a liquid in good yield. (9-Hexylcarbazole-3-yl)boronic acid pinacol ester (**5**) was subjected to Suzuki–Miyaura reaction either with 2-bromopyridine-4-carbaldehyde (**6a**) or 2-bromopyridine-5-carbaldehyde (**6b**) in the presence of potassium carbonate and bis(triphenylphosphine)palladium(II) dichloride in tetrahydrofuran [2]. Upon repeated purification by chromatography, compounds **7a** and **7b** were obtained as liquids in good yields (Scheme 1). Compounds **7a** and **7b** were fully characterised by NMR, FTIR, MS, elemental analysis, TGA, DSC, CV, and absorption and emission spectroscopy. The data are given in Supporting Information File 1.

### Thermal properties

**Figure 1:**
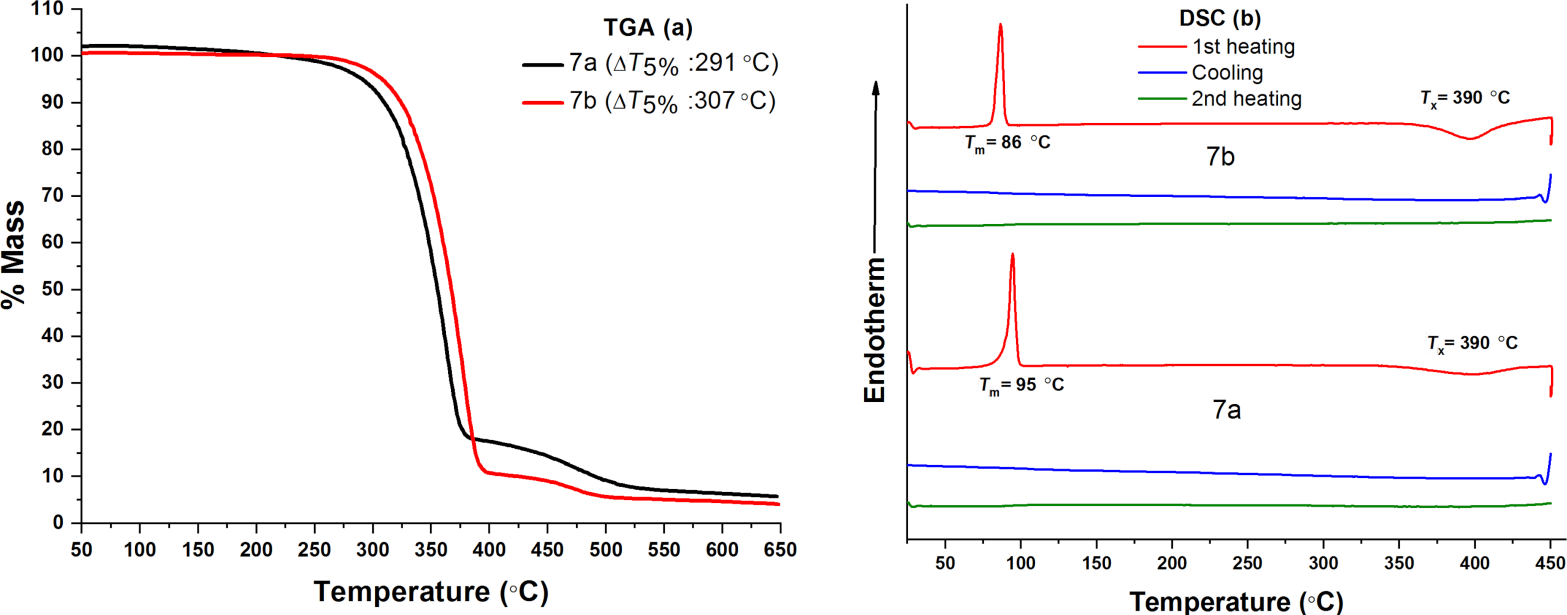
TGA (a) and DSC (b) curve of the compounds **7a** and **7b**.

The thermal properties of compounds **7a** and **7b** were investigated by thermogravimetric analyses (TGA) and differential scanning calorimetry (DSC). For TGA, compounds **7a** and **7b** were heated at 20 °C/min under nitrogen atmosphere. The decomposition temperatures (*T*_d_^5%^) corresponding to 5% weight losses for **7a** and **7b** were 291 °C and 307 °C, respectively. For DSC, compounds **7a** and **7b** were first heated to 450 °C and then cooled down to room temperature at 20 °C/min under a nitrogen atmosphere. Compounds **7a** and **7b** showed only clear melting transitions (*T*_m_) at 95 °C and 86 °C, respectively. Upon first cooling and second heating, no phase transitions were observed at all. TGA and DSC curves of compounds **7a** and **7b** are depicted in Figure 1. Thermal properties of compounds **7a** and **7b** are also summarised in Table 1.

**Table 1:** Thermal properties of compounds **7a** and **7b**.

compound	*T*_m_ (°C)	*T*_d_^5%^ (°C)

**7a**	95	291
**7b**	86	307

### Electrochemical properties

**Figure 2:**
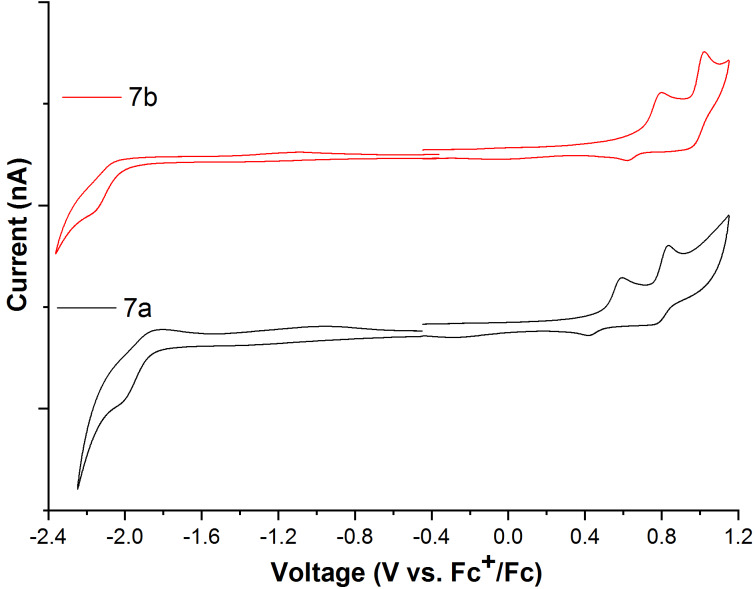
Cyclic voltammograms of compounds **7a** and **7b** in dichloromethane under argon atmosphere at room temperature.

The redox behaviour of compounds **7a** and **7b** was investigated by cyclic voltammetry in dichloromethane solution under argon atmosphere using tetrabutylammonium hexafluorophosphate as the electrolyte (Figure 2). A platinum disk was used as a working electrode, silver wire as the reference electrode and platinum wire as the counter electrode. The ferrocene-ferrocenium redox couple was used as an internal reference.

At positive potentials, compounds **7a** and **7b** exhibited two oxidation peaks; one is quasi-reversible at 0.55 V (**7a)** and 0.71 V (**7b**), and the other is reversible at 0.84 V (**7a**) and 0.99 V (**7b**). At negative potentials, compounds **7a** and **7b** only exhibited an irreversible reduction peak at −1.86 V and −1.93 V, respectively. The highest occupied molecular orbital and the lowest unoccupied molecular orbital energy levels (*E*_HOMO_, *E*_LUMO_) of compounds **7a** and **7b** were also calculated from the half-way anodic oxidation and onset cathodic reduction peak potentials, with respect to the energy level of ferrocene (4.8 eV below vacuum level) [38] by using the following equations; [39] *E*_HOMO_= −(4.8 + *E*_1/2_^ox^) and *E*_LUMO_= −(4.8 + *E*_onset_^red^). The HOMO–LUMO energy gap was calculated both from electrochemical data using Equation 1 and from optical data using Equation 2 [25,40–41].

[1] $$E_{\text{g}}{}^{\text{elec}}=\;E_{\text{LUMO}}-E_{\text{HOMO}}$$

[2] $$E_{\text{g}}{}^{\text{abs}}=\;1240/\lambda_{\text{onset}}{}^{\text{abs}}$$

**Figure 3:**
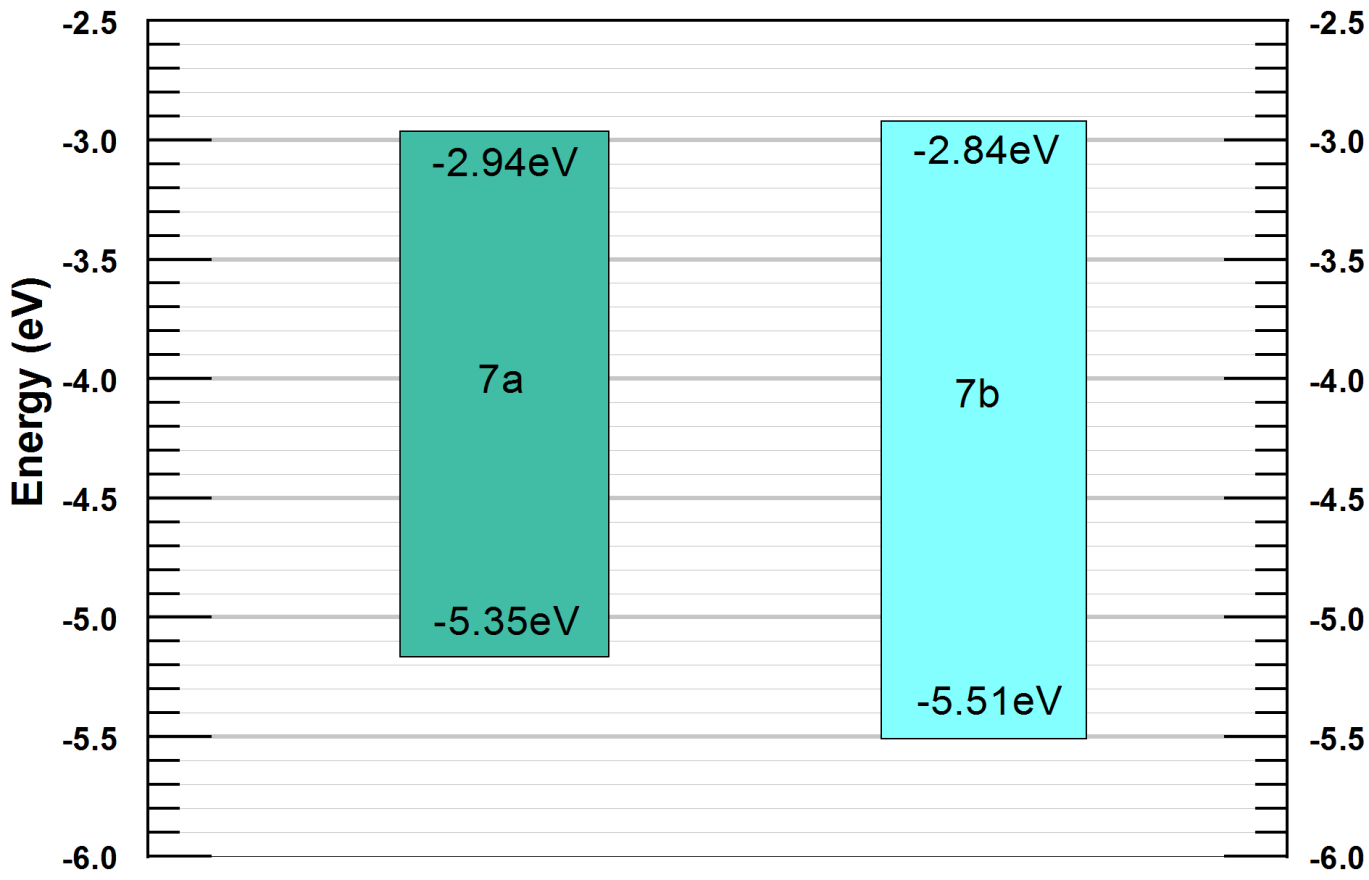
Energy levels of compounds **7a** and **7b**.

The optical energy gap (*E*_g_^o^) was higher than the electrochemical energy gap (*E*_g_^e^) for compounds **7a** and **7b**. The oxidation and reduction potentials and the HOMO–LUMO energy levels of both compounds are summarised in Table 2 and the energy levels are depicted in Figure 3.

**Table 2:** Oxidation, reduction and energy gap of compounds **7a** and **7b**.

compound	*E*_1/2_^ox^ (V)	*E*_onset_^red^ (V)	*E*_LUMO_ (eV)	*E*_HOMO_ (eV)	*E*_g_^e^/*E*_g_^o^ (eV)

**7a**	0.55, 0.84	−1.86	−2.94	−5.35	2.41/2.90
**7b**	0.71, 0.99	−1.96	−2.84	−5.51	2.68/2.99

### Optical properties

The absorption properties of compounds **7a** and **7b** were investigated in dichloromethane using a Duetta Fluorescence and Absorbance Spectrometer. Each compound (**7a**/**7b**) displayed three distinctive absorption bands in the UV–vis spectra: high energy bands and mid energy bands were assigned to π–π* and n–π* transitions, whereas the low energy bands were assigned to an intramolecular charge transfer (ICT) transition. The ICT band of **7b** at 373 nm was more intense than the ICT band of **7a** at 378 nm. This observation confirms that conjugation enhances ICT band intensity [42]. In **7b**, the formyl group is at the *para* position to the carbazole ring, thus giving rise to conjugation. In **7a**, however, the formyl group is at the *meta* position to the carbazole ring.

**Figure 4:**
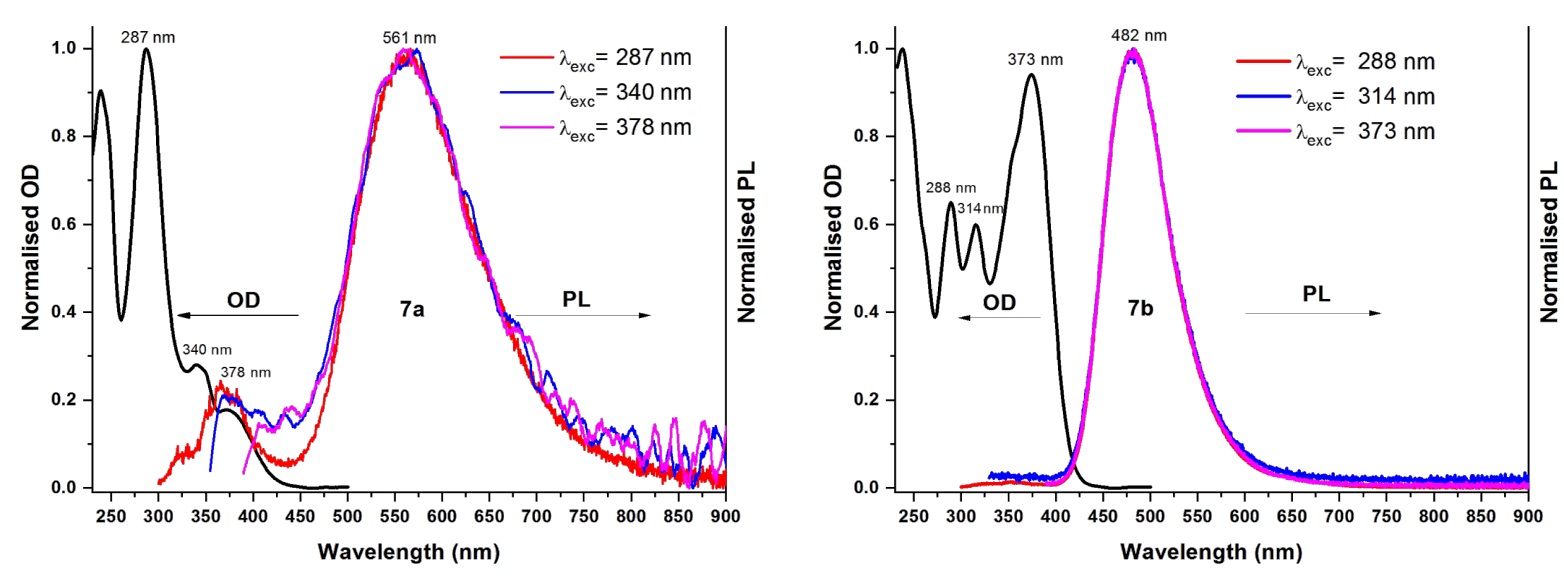
Normalised UV–vis and PL spectra of compounds **7a** and **7b** in dichloromethane.

The photoluminescence (PL) properties of compounds **7a** and **7b** were investigated in dichloromethane using a Duetta Fluorescence and Absorbance Spectrometer. Compounds **7a** and **7b** gave emission maxima at 561 nm and 482 nm, respectively. The UV–vis and PL spectra of the compounds are given in Figure 4.

### Solvatochromism

**Figure 5:**
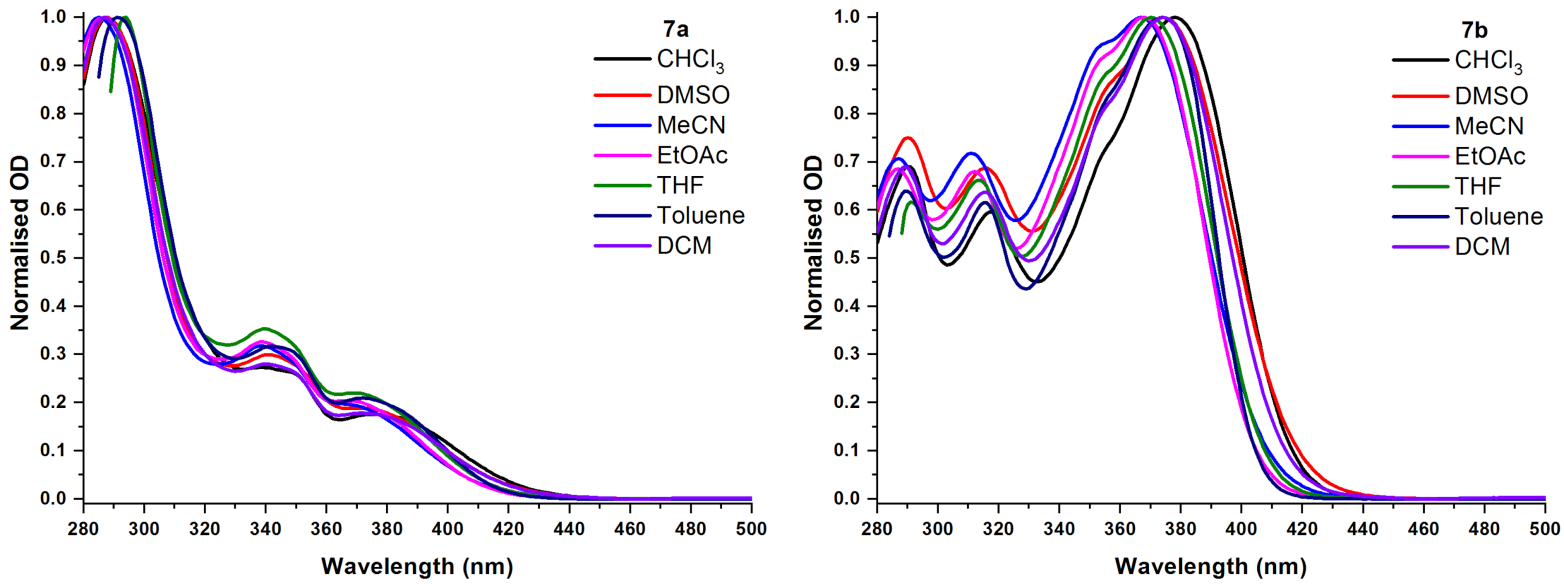
Normalised UV–vis spectra of compounds **7a** and **7b** in different solvents.

In general, ICT-based absorption and emission bands show solvent dependency. This is better known as solvatochromism. The ICT behaviour of compounds **7a** and **7b** was further investigated in different solvents. Normalised UV–vis spectra of compounds **7a** and **7b** in different solvents are depicted in Figure 5. The spectral profiles remained almost unchanged in different solvents, but there is greater variance in the spectra of compound **7b**.

**Figure 6:**
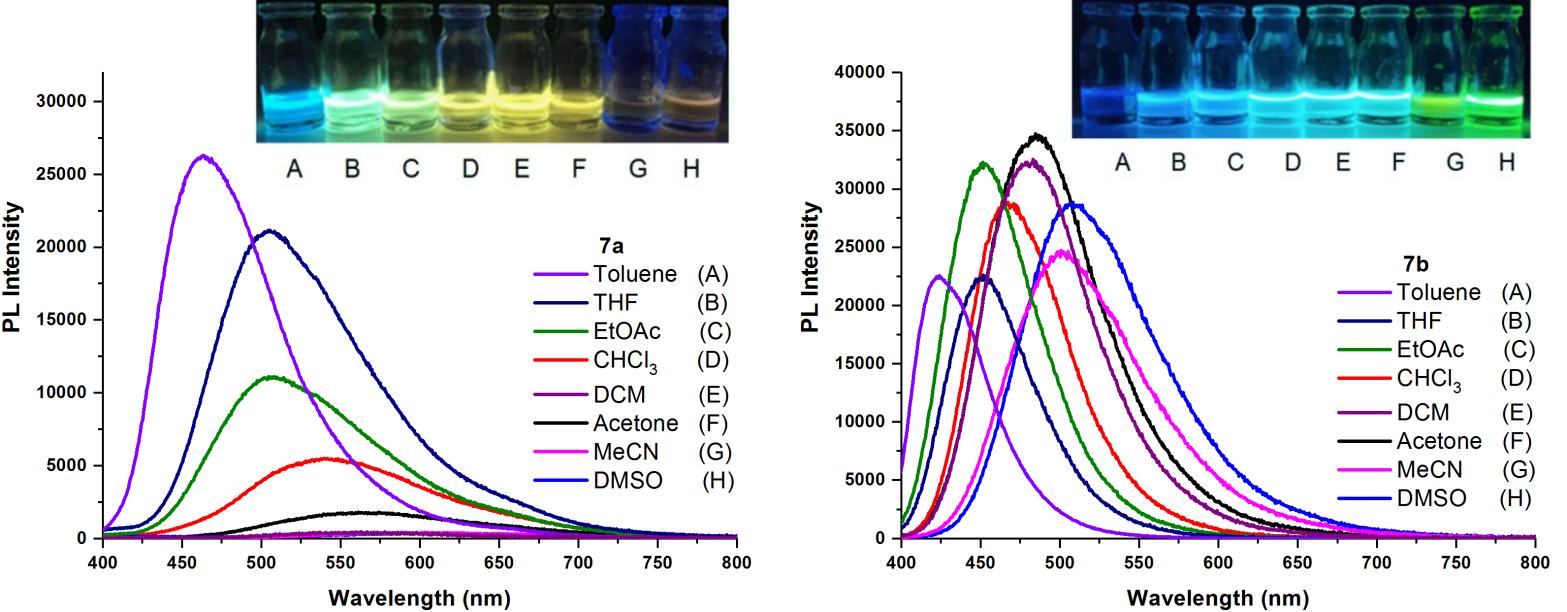
PL spectra of compounds **7a** and **7b** in different solvents (A–H).

The PL spectra of compounds **7a** and **7b** displayed either dual emission bands or a single emission band. This was dependent on the excitation wavelength chosen and the solvent used. It is believed that the dual emission was due to mixed locally excited (LE) and intramolecular charge transfer (ICT) states and the single emission was due to the ICT state. Photoluminescence (PL) spectra of compounds **7a** and **7b** in different solvents are shown in Figure 6.

Upon excitation at π–π*/n–π* bands (λ_exc_= 245–349 nm for **7a** and 248–356 nm for **7b**), the PL spectra of compound **7a** in most solvents depicted dual emission bands, one from the locally excited state and one from ICT. On the other hand, the PL spectra of compound **7b** in most solvents interestingly depicted only a single emission band from ICT. Upon excitation at the ICT band (λ_exc_ = 370–377 nm for **7a** and 367–378 nm for **7b**), only a single emission band was observed for both compounds (**7a** and **7b**). As seen in Figure 6, a red-shift was observed in the emission maxima as the microscopic solvent polarity [43–44], *E*_T_(30), increased from toluene to dimethyl sulfoxide (see also Table 3). A 141 nm red-shift was observed for **7a** (from 465 nm to 606 nm) and an 86 nm red-shift was observed for **7b** (from 423 nm to 509 nm). In comparison for **7b**, this red-shift was more pronounced for **7a**. This indicates that the excited state dipole moment is much greater than the ground state dipole moment.

**Table 3:** Maximum emission wavelength (λ_em_^max^) of compounds **7a** and **7b** under different solvents.

entry	solvents, E_T_(30)	**7a** (λ_em_, nm)	**7b** (λ_em_ ,nm)

A	toluene, 33.0	465	423
B	tetrahydrofuran, 37.4	505	452
C	ethyl acetate, 38.1	509	452
D	chloroform, 39.1	543	467
E	dichloromethane, 40.7	561	482
F	acetone, 42.2	564	486
G	acetonitrile, 45.6	600	501
H	dimethyl sulfoxide, 45.1	606	509

### Quantum yields

The relative fluorescent quantum yields (ϕ_FL_) of compounds **7a** and **7b** were determined in dichloromethane by using rhodamine B (ϕ_FL_ = 49% at λ_exc_=355 nm) in ethanol as reference [45]. ϕ_FL_ of compounds **7a** and **7b** was 5.4% and 97.4%, respectively. An estimated error in quantum yield calculations is ca. 10%. The details of the calculations are given in Supporting Information File 1. Surprisingly, compound **7b**, in which the formyl group is at the *para* position to the carbazole ring, was much more emissive than compound **7a**, in which the formyl group is at the *meta* position to the carbazole ring.

## Conclusion

In this work, two novel compounds **7a** and **7b** were successfully synthesised in good yields and demonstrated good thermal stability. Compounds **7a** and **7b** showed intramolecular charge transport properties with positive solvatochromism. Whilst **7a** showed very low emission intensity, **7b** showed very high emission intensity. It is noted that the conjugation in compound **7b** encompasses the N atom of the carbazole ring and the formyl functionality (viz. the donor/acceptor units of the ICT component), whereas the link by conjugation between the same functionalities in **7a** is missing. The resulting stronger ICT component in **7b** explains the big difference in photophysical properties.

## Experimental

All reagents were standard reagent grade and purchased from Sigma-Aldrich, Merck and Alfa Aesar. Inert reactions were performed under an argon atmosphere. Nuclear magnetic resonance (NMR) spectra were obtained on an Agilent Premium Compact NMR spectrometer (600 MHz for ^1^H NMR, 150 MHz for ^13^C NMR) with tetramethylsilane as internal standard. Elemental analysis was performed on a Costech Elemental system. The IR spectra were obtained (4000–400 cm^−1^) using a Shimadzu IRAffinity-1S Fourier transform infrared spectrophotometer. The mass spectra were obtained by Bruker microTOFq mass spectrometers to obtain low- and high-resolution spectra using electron ionisation (EI) or electrospray ionisation (ESI) techniques. UV, PL and photoluminescence quantum yields were measured on a Duetta two-in-one fluorescence and absorbance spectrometer from Horiba Scientific. Both absorption and emission solutions for reference and samples had a concentration of 10^−6^ M. CV measurements were obtained using a CH Instruments 602E electrochemical workstation with iR compensation using dry dichloromethane. Thermogravimetric analysis was conducted using a Netzsch TG 209 F3 Tarsus Thermogravimetric Analyser under a constant flow of nitrogen. Differential scanning calorimetry was determined on a Netzsch DSC 214 Polyma instrument.

**3-Bromocarbazole (2): 2** was synthesised as reported previously [32–33]. A solution of *N*-bromosuccinimide (1.1 g, 5.98 mmol) in dimethylformamide was added dropwise to a solution of carbazole (**1**, 1 g, 5.96 mmol) in dimethylformamide (15 mL) at 0 °C. The reaction mixture was then stirred at room temperature for 24 h. The reaction was poured into distilled water to give a cream coloured precipitate. The precipitate was filtered off under vacuum and washed with distilled water (3 × 20 mL). The precipitate was dissolved in ethyl acetate, dried with sodium sulfate and filtered. Upon concentration under reduced pressure the crude product was obtained as a brown solid. After crystallisation of the crude product with chloroform, the pure product **2** (692 mg, 47%) was obtained as white crystals. *R*_f_ (ethyl acetate/hexane, 1:6 v/v): 0.43; melting point: 200–201°C; ^1^H NMR (600 MHz, CDCI_3_) δ (ppm) 8.19 (d, *J* = 1.9 Hz, 1H), 8.08 (s, 1H), 8.02 (dd, *J* = 7.7, 1.1 Hz, 1H), 7.50 (dd, *J* = 8.5, 1.9 Hz, 1H), 7.47–7.40 (m, 2H), 7.31 (d, *J* = 8.6 Hz, 1H), 7.25 (td, *J* = 6.3, 1.8 Hz, 1H).

**3-Bromo-9-hexylcarbazole (4): 4** was synthesised as reported previously [33–35]. A mixture of 3-bromocarbazole (**2**, 1.5 g, 6.0 mmol), 1-bromohexane (4.0 g, 24.3 mmol), tetrabutylammonium iodide (225 mg, 0.6 mmol) and aqueous sodium hydroxide (26 mL, 50%) was heated at 77 °C for 8 h. The product was extracted with dichloromethane (3 × 20 mL), and the combined extracts were dried over sodium sulfate and then filtered. Upon concentration under reduced pressure, the crude product was obtained as a light yellow liquid. The crude product was purified by flash column chromatography (2:98 triethylamine/hexane v/v). Pure compound **4** (1.7 g, 84%) was obtained as a colourless liquid which solidified on standing. *R*_f_ (hexane): 0.3, melting point: 48–49 °C; ^1^H NMR (600 MHz, CDCI_3_) δ (ppm) 8.20 (d, *J* = 1.9 Hz, 1H), 8.04 (d, *J* = 7.8, 1.1 Hz, 1H), 7.53 (dd, *J* = 8.6, 1.9 Hz, 1H), 7.48 (td, *J* = 8.3, 1.2 Hz, 1H), 7.40 (d, *J* = 8.2 Hz, 1H), 7.28 (d, *J* = 8.6 Hz, 1H), 7.24 (t, 1H), 4.27 (t, *J* = 7.3 Hz, 3H), 1.85 (p, *J* = 7.4 Hz, 3H), 1.34–1.23 (m, 6H), 0.86 (t, *J* = 7.0 Hz, 4H).

**(9-Hexylcarbazole-3-yl)boronic acid pinacol ester (5): 5** was synthesised as reported previously [36–37]. Bis(pinacolato)diboron (423 mg, 1.7 mmol), potassium acetate (446 mg, 4.5 mmol) and dichlorobis(triphenylphosphine)palladium(II) (35 mg, 0.05 mmol) catalyst were added to a 3-bromo-*N*-hexylcarbazole (**4**, 500 mg, 1.5 mmol) solution in 1,4-dioxane (15 mL). The reaction mixture was heated at 90 °C for 24 h under an argon atmosphere. The crude product was extracted with dichloromethane (3 × 20 mL), and the combined extracts were dried over sodium sulfate and then filtered. Upon concentration under reduced pressure, the crude product was obtained as a brown–black liquid. The product was purified by flash column chromatography (CH_2_Cl_2_/hexane 1:4 v/v). Pure product **5** (404 mg, 71%) was obtained as a colourless liquid. *R*_f_ (CH_2_Cl_2_/hexane, 1:2 v/v): 0.32, ^1^H NMR (600 MHz, CDCI_3_) δ (ppm) 8.60 (s, 1H), 8.13 (d, *J* = 7.6 Hz, 1H), 7.91 (dd, *J* = 8.1, 2.5 Hz, 1H), 7.46 (t, *J* = 7.4 Hz, 1H), 7.42–7.36 (m, 2H), 7.23 (d, *J* = 7.6 Hz, 1H), 4.30 (t, *J* = 7.0 Hz, 2H), 1.86 (t, *J* = 7.6 Hz, 2H), 1.49–1.15 (m, 18H), 0.85 (t, *J* = 7.1 Hz, 3H).

**Compound 7a:** (9-Hexylcarbazol-3-yl)boronic acid pinacol ester (**5**, 447 mg, 1.2 mmol), 2-bromopyridine-4-carbaldehyde (**6a**, 147 mg, 0.8 mmol), potassium carbonate (1 M, 9.6 mL) and dichlorobis(triphenylphosphine)palladium(II) (40 mg, 0.06 mmol) were dissolved in tetrahydrofuran (20 mL). The reaction mixture was refluxed for 24 h under an argon atmosphere. After removing the solvent, the crude product was dissolved in dichloromethane and washed with water (3 × 20 mL). The combined extracts were dried over sodium sulfate and then filtered. Upon concentration under reduced pressure, the crude product was obtained as a dark green–yellow liquid. The crude product was first purified by flash column chromatography (1:3 CH_2_Cl_2_/hexane v/v) followed by preparative thin-layer chromatography (4:2:1 hexane/CHCI_3_/MeOH v/v/v). The pure product **7a** (176 mg, 63%) was obtained as a yellow liquid which solidified on standing. *R*_f_ (dichloromethane/hexane, 1:1 v/v): 0.3, melting point: 91–93 °C, ^1^H NMR (600 MHz, CDCI_3_) δ (ppm) 10.17 (s, 1H), 8.95 (dd, *J* = 4.8, 0.9 Hz, 1H), 8.83 (d, *J* = 1.8 Hz, 1H), 8.24 (s, 1H), 8.23–8.16 (m, 3H), 7.58 (dd, *J* = 4.9, 1.4 Hz, 1H), 7.53–7.46 (m, 3H), 7.44 (d, *J* = 8.0 Hz, 1H), 7.28 (dt, *J* = 7.3, 0.8 Hz, 1H), 4.33 (t, *J* = 7.3 Hz, 3H), 1.90 (p, *J* = 7.7 Hz, 3H), 1.47–1.37 (m, 3H), 1.37–1.19 (m, 6H), 0.87 (t, *J* = 7.1 Hz, 4H); ^13^C NMR (600 MHz, CDCI_3_) δ (ppm) 192.0, 160.0, 150.9, 142.5, 141.5, 141.0, 129.1, 126.1, 124.8, 123.4, 123.1, 120.6, 119.4, 119.3, 119.3, 118.6, 109.0, 108.9, 43.3, 31.6, 29.0, 27.0, 22.5, 14.0; FTIR (cm^−1^): 2956, 2924, 2911, 2872, 2851, 1697; anal. calcd for C_24_H_24_N_2_O, C: 80.87, H: 6.79, N: 7.86; found: C: 80.86, H: 6.92, N: 7.57.

**Compound 7b:** (9-Hexylcarbazol-3-yl)boronic acid pinacol ester (**5**, 144 mg, 0.4 mmol), 2-bromopyridine-5-carbaldehyde (**6b**, 47 mg, 0.3 mmol), potassium carbonate (1 M, 3 mL) and dichlorobis(triphenylphosphine)palladium(II) (13 mg, 0.02 mmol) were dissolved in tetrahydrofuran (10 mL). The reaction mixture was refluxed for 6 h under an argon atmosphere. After removing the solvent, the crude product was dissolved in dichloromethane and washed with water (3 × 20 mL). The combined extracts were dried over sodium sulfate and then filtered. Upon concentration under reduced pressure, the crude product was obtained as a dark green-yellow liquid. The crude product was first purified by flash column chromatography (dichloromethane/hexane, 1:1 v/v) followed by preparative thin-layer chromatography (hexane/chloroform/MeOH, 10:5:2 v/v/v). The pure product **7b** (67 mg, 75%) was obtained as a yellow solid. *R*_f_ (dichloromethane/hexane, 5:1 v/v): 0.3; melting point: 89–91°C; ^1^H NMR (600 MHz, CDCI_3_) δ (ppm): 10.12 (s, 1H), 9.13 (d, *J* = 2.2 Hz, 1H), 8.88 (d, *J* = 1.8 Hz, 1H), 8.26–8.17 (m, 3H), 8.01 (d, *J* = 8.3 Hz, 1H), 7.53–7.48 (m, 2H), 7.44 (d, *J* = 8.1 Hz, 1H), 7.29 (t, *J* = 7.4 Hz, 1H), 4.34 (t, *J* = 7.3 Hz, 3H), 1.90 (p, *J* = 7.4 Hz, 2H), 1.44–1.25 (m, 6H), 0.87 (t, *J* = 7.1 Hz, 3H); ^13^C NMR (600 MHz, CDCI_3_) δ (ppm) 190.5, 163.1, 152.7, 142.0, 141.1, 136.2, 129.1, 128.8, 126.2, 125.3, 123.5, 123.1, 120.7, 120.1, 119.9, 119.5, 109.1, 109.1, 43.3, 31.5, 28.9, 26.9, 22.5, 14.0; FTIR (cm^−1^): 2950, 2924, 2866, 2852, 2822, 2786, 2724, 1696, anal. calcd for: C_24_H_24_N_2_O, C: 80.87, H: 6.79, N: 7.86; found C: 80.88, H: 6.91, N: 7.12; MS (EI+, *m*/*z*): 356 (M^+^, 73%), 285 ([M − C_5_H_11_]^+^, 100%), HRMS (FAB^+^, *m*/*z*): calculated for C_24_H_24_N_2_O [M]^+^ 356.1889, found for [M]^+^ 356.1893 (error: +1.2 ppm).

## Supporting Information

The Supporting Information features the followings: 1) ^1^H NMR and ^13^C NMR spectra; 2) FTIR spectra; 3) mass and HRMS spectra; 4) calculations of relative fluorescence quantum yields.

File 1 NMR, FTIR, MS and HRMS spectra of compounds and relative quantum yield calculations.
