# Supplementary material for: Synthesis of novel multifunctional carbazole-based molecules and their thermal, electrochemical and optical properties
Source: Beilstein J Org Chem. 2020 May 19;16:1066–74. doi: 10.3762/bjoc.16.93 (PMC7277623; doi:10.3762/bjoc.16.93)
Supplement: File 1 — NMR, FTIR, MS and HRMS spectra of compounds and relative quantum yield calculations. [file Beilstein_J_Org_Chem-16-1066-s001.pdf]

BEILSTEIN JOURNAL OF ORGANIC CHEMISTRY

Supporting Information

for

Synthesis of novel multifunctional carbazole-based molecules and their thermal, electrochemical and optical properties

Nuray Altinolcek, Ahmet Battal, Mustafa Tavasli, William J. Peveler, Holly A. Yu and Peter J. Skabara

Beilstein J. Org. Chem. **2020,** *16,* 1066–1074. doi:10.3762/bjoc.16.93

NMR, FTIR, MS and HRMS spectra of compounds and relative quantum yield calculations

License and Terms: This is a supporting information file under the terms of the Creative Commons Attribution License (http://creativecommons.org/licenses/by/4.0). Please note that the reuse, redistribution and reproduction in particular requires that the authors and source are credited.
The license is subject to the *Beilstein Journal of Organic Chemistry* terms and conditions: (https://www.beilstein-journals.org/bjoc)

Table of contents

1. ^{1}H and ^{13}C NMR spectra	Figure S1:	^{1}H NMR spectrum of 3-bromocarbazole (**2**)	S3
	Figure S2:	^{1}H NMR spectrum of 3-bromo-*N*-hexylcarbazole (**4**)	S3
	Figure S3:	^{1}H NMR spectrum of *N*-hexylcarbazole-3-yl-boronic acid pinocolate ester (**5**)	S4
	Figure S4:	^{1}H NMR spectrum of compound **7a**	S4
	Figure S5:	^{13}C NMR spectrum of compound **7a**	S5
	Figure S6:	^{1}H NMR spectrum of compound **7b**	S5
	Figure S7:	^{13}C NMR spectrum of compound **7b**	S5
2. FTIR spectra	Figure S8:	FTIR spectrum of compounds **7a** and **7b**	S6
3. Mass and HRMS spectra	Figure S9:	Mass spectrum of compound **7b**	S6
	Figure S10:	HRMS result of compound **7b**	S6
4. Calculations of relative fluorescence quantum yields	Figure S11:	Absorption at 355 nm-integrated emission of samples (**7a** and **7b**) and reference (rhodamine B)	S7
	Table S1:	Optical density and integrated PL intensity values of reference (rhodamine B) and samples (**7a** and **7b**)	S8
	Figure S12:	Absorption spectra of rhodamine B, **7a** and **7b**	S8
	Figure S13:	Emission (PL) spectra of rhodamine B, **7a** and **7b**, excited at 355 nm	S8

1. ^{1}H and ^{13}C NMR spectra

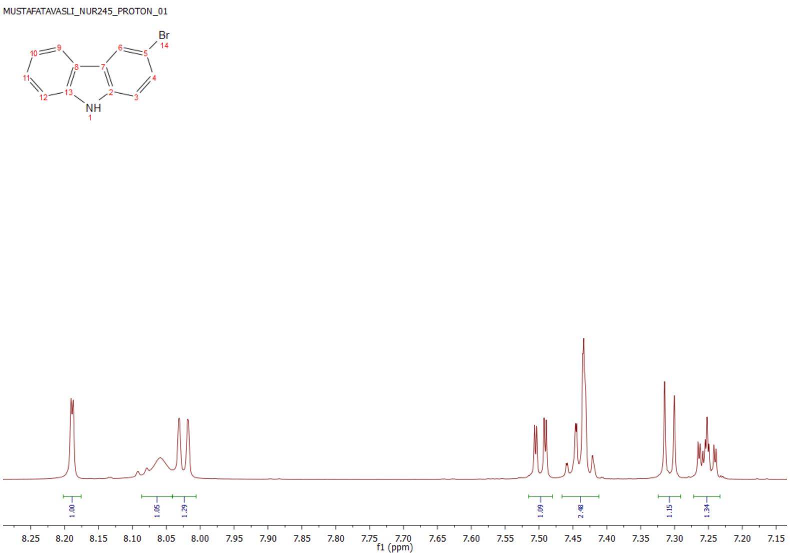

Figure S1: ^{1}H NMR spectrum of 3-bromocarbazole (**2**).

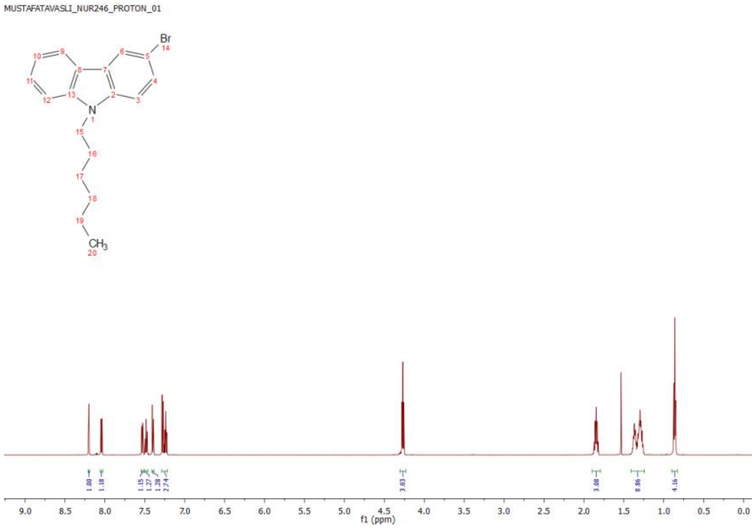

Figure S2: ^{1}H NMR spectrum of 3-bromo-N-hexylcarbazole (**4**).

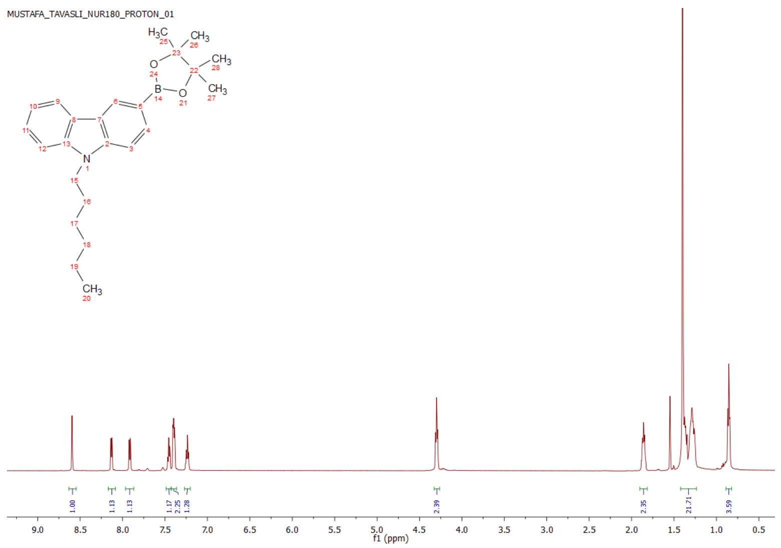

Figure S3: ^{1}H NMR spectrum of *N*-hexylcarbazole-3-yl-boronic acid pinocolate ester (**5**).

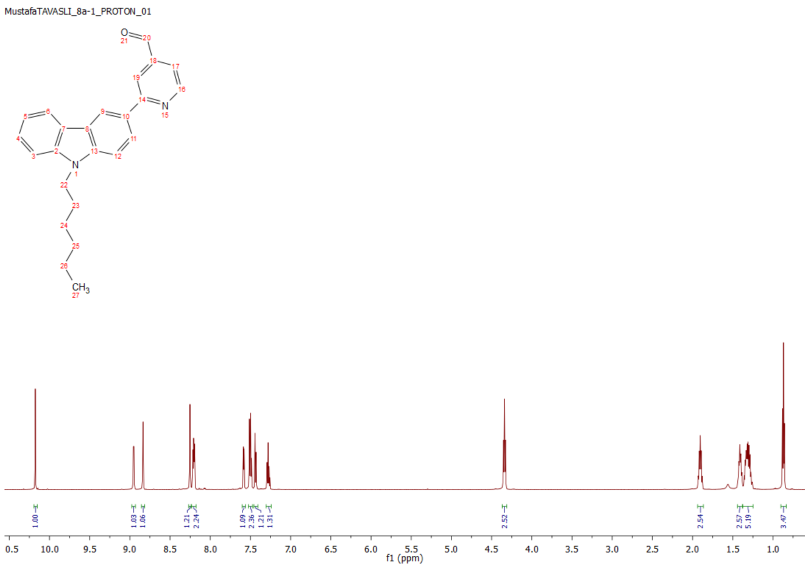

Figure S4: ^{1}H NMR spectrum of compound **7a**.

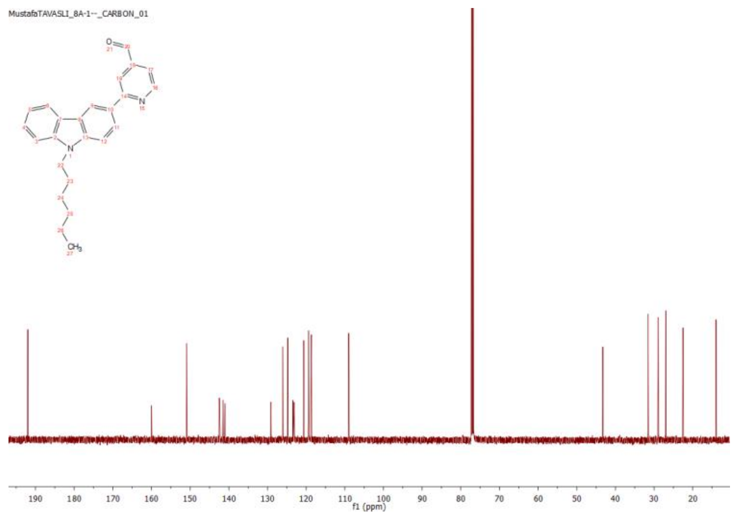

Figure S5: ^{13}C NMR spectrum of compound **7a**.

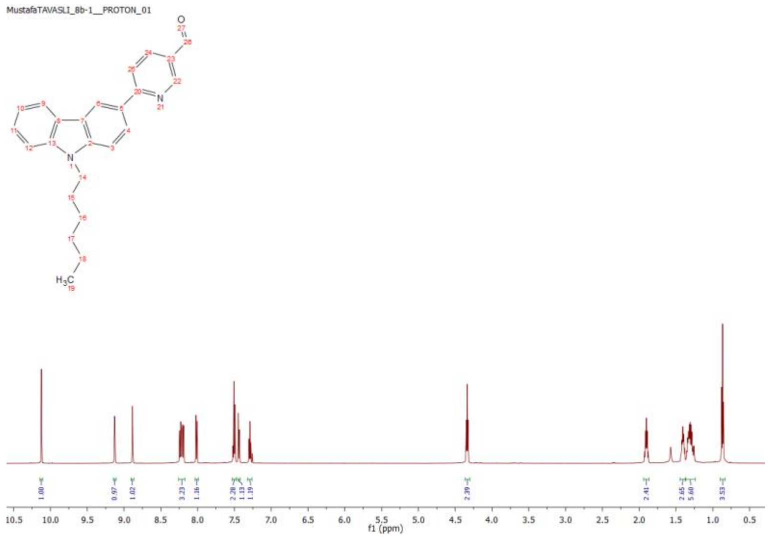

Figure S6: ^{1}H NMR spectrum of compound **7b**.

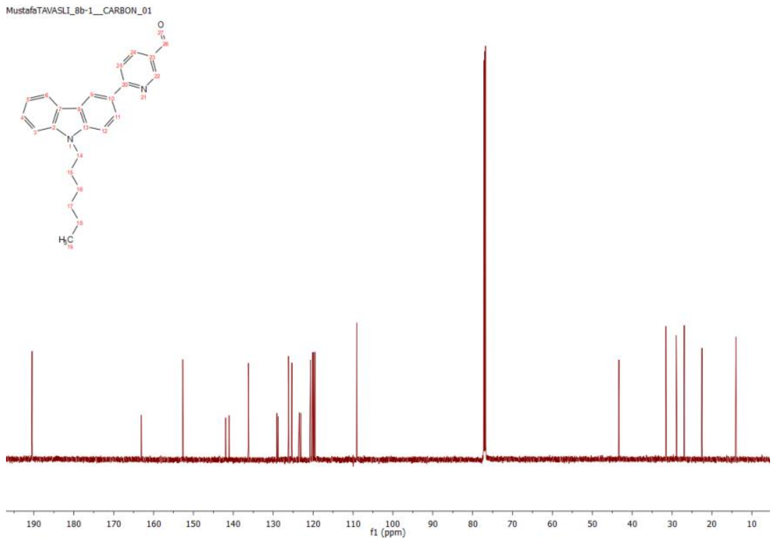

Figure S7: ^{13}C NMR spectrum of compound **7b**.

2. FTIR spectra

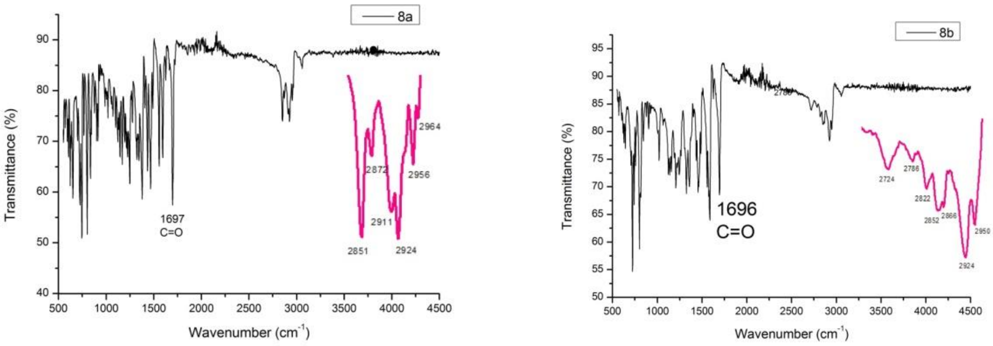

Figure S8: FTIR spectrum of compounds **7a** and **7b**

3. Mass and HRMS spectrum

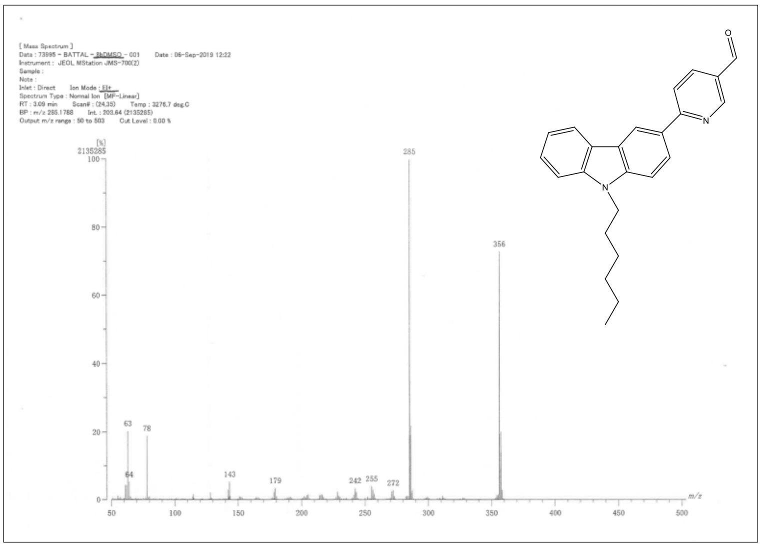

Figure S9: Mass spectrum of compound **7b**.

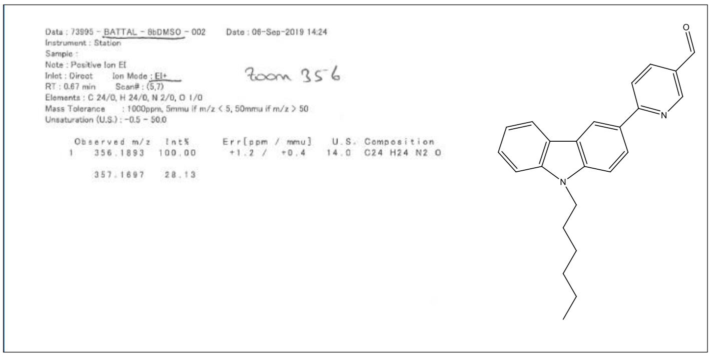

Figure S10: HRMS result of compound **7b**.

4. Calculations of relative fluorescence quantum yields

Relative fluorescent quantum yields of samples (ϕ_S) were calculated from Figure S11 by using equation 1, where y_S is the integrated fluorescence intensity of samples (**7a**/**7b**), y_R is the integrated fluorescence intensity of reference (Rhodamine B), n is the refractive index of solvents used.

$$\phi_s = y_S . \frac{\phi_R}{y_R} . \frac{{n_s}^2}{{n_r}^2} \qquad (1)$$

Figure S11, plotted from Table S1, depicts absorption at 355 nm (OD, optical density) versus integrated emission (integrated fluorescence intensity of reference and samples).

For this, samples (**7a** and **7b**) were dissolved in dichloromethane (*n* = 1.435) and reference (rhodamine B) was dissolved in absolute ethanol (*n* = 1.361). Different concentrations of reference (C1–C7) and samples (**7a**: C1–C5 and **7b**: C1–C8) were then prepared. For each concentration absorption spectra of reference and samples were recorded as depicted in Figure S12. Both reference and samples were excited at 355 nm, then emission spectra of reference and samples were recorded as depicted in Figure S13. All of these values are summarised in Table S1.

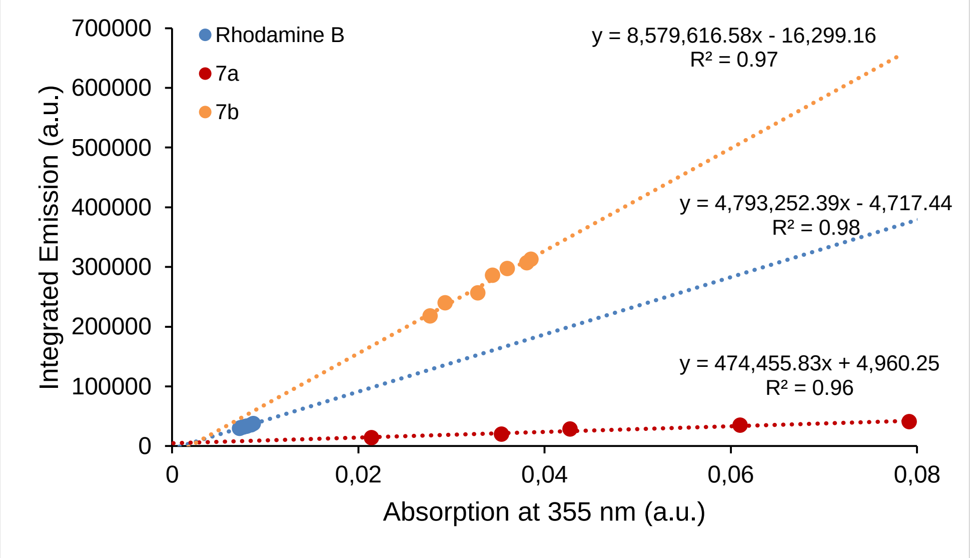

Figure S11: Absorption at 355 nm-integrated emission of samples (**7a** and **7b**) and reference (rhodamine B)

Table S1: Optical density and integrated PL intensity values of reference (rhodamine B) and samples (7a and 7b)								
	**Rhodamine B** (λ_{exc}= 355 nm)			**7a** (**λ_{exc}= 355 nm**)			**7b** (**λ_{exc}= 355 nm**)	
Unsorted	OD (a.u)	Integrated PL intensity (a.u)	Unsorted	OD (a.u)	Integrated PL intensity	Unsorted	OD (a.u)	Integrated PL intensity
C1	0.00873	37591.15917	C1	0.02143	14075.26276	C1	0.03855	312854.9439
C2	0.00849	35459.07801	C2	0.03538	20063.43206	C2	0.03810	307049.5385
C3	0.00804	33547.29896	C3	0.04274	28371.31840	C3	0.03599	297563.9041
C4	0.00786	33095.41848	C4	0.06100	35022.74323	C4	0.03441	286229.5682
C5	0.00762	31942.84488	C5	0.07916	41000.28228	C6	0.03284	256749.5341
C6	0.00734	30932.30389				C7	0.02933	240013.4747
C7	0.00726	29668.39935				C8	0.02770	218127.6961
**Gradient=PL/OD**		4,793,252.39	**Gradient=PL/OD**		474,455.83	**Gradient=PL/OD**		8,579,616.58
**Refractive index of ethanol**		1.361	**Refractive index of dichloromethane**		1.435	**Refractive index of dichloromethane**		1.435
**PLQY**	0.49		**PLQY**	0.054		**PLQY**	0.974	
**Unit**	1.02227E-07		Estimated error on values ca. ± 10% RSD.					

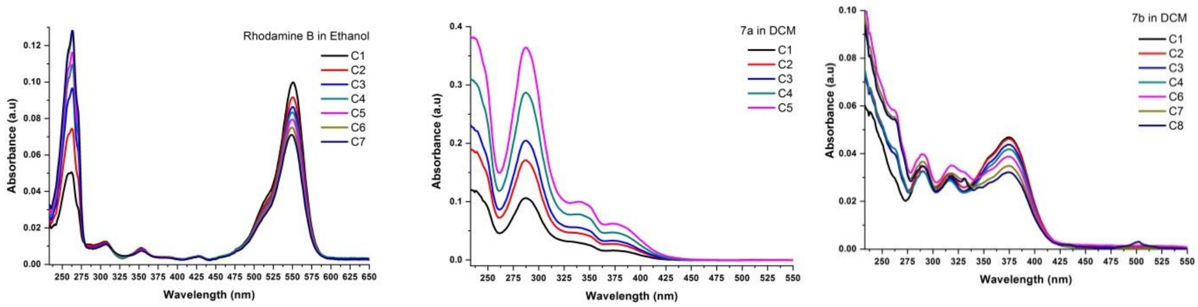

Figure S12: Absorption spectra of rhodamine B, **7a** and **7b**.

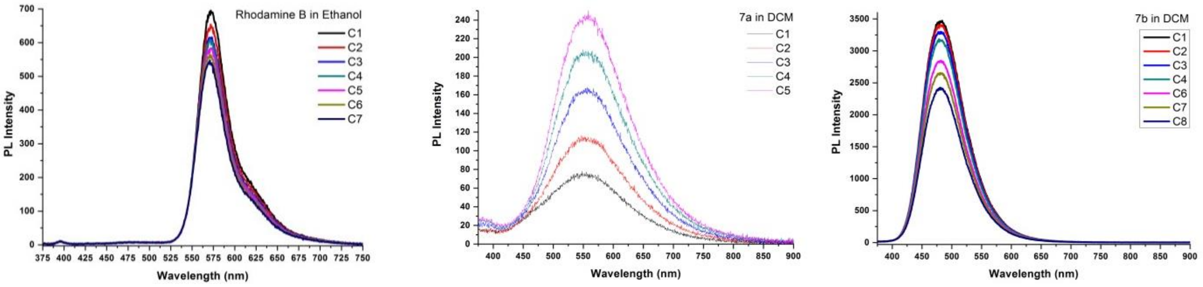

Figure S13: Emission (PL) spectra of rhodamine B, **7a** and **7b**, excited at 355 nm.
